# Driving Sustainable Innovation through Digital Transformation: A Behavioural Model of the Upcycling Craft Industry in Indonesia

**DOI:** 10.12688/f1000research.174640.2

**Published:** 2026-05-05

**Authors:** Faza Wahmuda, Ellya Zulaikha, Hadziq Fabroyir

**Affiliations:** 1 Interdisciplinary School of Management Technology, Institut Teknologi Sepuluh Nopember, Surabaya, East Java, Indonesia; 2 Institut Teknologi Adhi Tama Surabaya, East Java, Indonesia; 3 Industrial Design Department, Institut Teknologi Sepuluh Nopember, Surabaya, East Java, Indonesia; 4 Informatics Department, Institut Teknologi Sepuluh Nopember, Surabaya, East Java, Indonesia

**Keywords:** Digital Transformation, Upcycling Craft Industry, Behavioral-Based Digital Transformation (BBDT), Innovation.

## Abstract

**Background:**

Digital transformation has emerged as a key driver of competitiveness across industries, yet its adoption in the upcycling craft sector remains insufficiently explored. Existing studies often focus on large enterprises, leaving a research gap in understanding how micro-scale, resource-constrained upcycling businesses integrate digital technologies, specifically within the Indonesian upcycling craft sector context. This study addresses this gap by developing the Behavioral-Based Digital Transformation (BBDT) model to investigate behavioral determinants of digital adoption, implementation, and innovation outcomes in Indonesia’s sustainability-oriented creative sector.

**Method:**

This study employed a sequential exploratory mixed-methods approach. The qualitative phase involved field observations and in-depth interviews with three upcycling craft enterprises, while the quantitative phase surveyed 30 micro-scale upcycling businesses across multiple Indonesian regions. Data were analyzed using thematic coding, NVivo software, and Partial Least Squares Structural Equation Modeling (PLS-SEM) to test the proposed Behavioral-Based Digital Transformation (BBDT) model.

**Results and Conclusion:**

Findings indicate that internal and external factors, including leadership commitment, financial capability, and market demand, significantly shape behavioral orientation toward digital adoption. Behavioral intentions, in turn, drive digital technology implementation, enhancing operational efficiency, material optimization, and product innovation. Digital adoption acts as both an outcome and enabler of early-stage sustainable transformation pathways, sustainable transformation, with innovation reinforcing initial cycles of engagement with technology. The study provides preliminary support for the mediating role of behavioral intention and underscores the critical importance of human and organizational readiness. These insights offer theoretical contributions to TAM, TPB, and IDT integration and practical guidance for policymakers, creative communities, and micro-enterprise owners.

## 1. Introduction

The circular economy approach has emerged as a strategic initiative in pursuing renewable production and consumption systems, playing a fundamental role in promoting environmental preservation within business practices ( Civera et al., 2025; Perotti et al., 2024). More specifically, in the context of business management, the circular economy refers to the implementation of practices aimed at maximizing resource efficiency, reducing waste and pollution, and supporting environmental sustainability ( Centobelli et al., 2020; Salvador et al., 2020). In this vein, upcycling has been introduced as a component of the circular economy, repurposing used materials to create high-value and high-quality products ( Schulte et al., 2025). Unlike conventional recycling practices, upcycling is a creative engineering process that integrates design innovation, aesthetic value, environmental awareness, and resource efficiency into a sustainable production system ( Schulte et al., 2025). Furthermore, within the circular economy literature, upcycling activities conducted by individuals or small and medium-sized enterprises (SMEs) are found to have a greater impact than industrial-scale upcycling ( Singh, 2022; Singh et al., 2019; Sung et al., 2019). Given the growing concern over increasing waste generation, upcycling has once again become an increasingly important theme in the circular economy paradigm.

In this regard, several studies have linked business commitment, which gradually translates sustainability objectives into business practices, with the emergence of circular business models ( Aranda-Usón et al., 2020; Perotti et al., 2024). Furthermore, as the circular economy ecosystem has not yet become a dominant model, Aiello et al. (2025) noted that circular processes heavily depend on technical and industrial innovation practices, requiring continuous technological advancement and digitalization. Nevertheless, digital technologies provide new opportunities for SMEs to drive innovation and implement circular economy initiatives, transforming their business orientation toward environmentally friendly practices while achieving high profitability ( Chaudhuri et al., 2022). This transformation involves the strategic adoption and integration of digital technologies across all business operations to enhance efficiency, expand market reach, and strengthen competitiveness ( Matt et al., 2015; Roux et al., 2023). Previous research has investigated the interplay between digital technologies and the circular economy in the context of SMEs (e.g., Hassoun et al., 2022; Kerdlap et al., 2019; Srisathan et al., 2025; Zheng et al., 2025). Findings from these studies indicate that digital technologies enhance resource efficiency and reduce waste, demonstrating strong potential for achieving business sustainability ( Hassoun et al., 2022; Srisathan et al., 2025; Zheng et al., 2025). However, Kerdlap et al. (2019) highlighted that digital technology adoption can lead to high implementation costs and technical capability limitations, particularly in developing countries.

The extent to which digital technology adoption is implemented within business operational activities and its influence on innovation and performance remains debated. The circular economy literature also identifies behavioral and organizational barriers that hinder the effective implementation of digital technologies, including a lack of clarity in vision and strategic direction related to technology roadmapping ( Ivančić et al., 2019; Zheng et al., 2025) and resistance to change when adopting new technologies ( Ivančić et al., 2019; Nylén & Holmström, 2015; Srisathan et al., 2025). Meanwhile, Wahmuda et al. (2024), in a systematic literature review, revealed that digital technology adoption remains weak. On the other hand, Turulja and Bajgoric (2019), from the perspective of contingency theory, argue that the development of digital technologies serves as a driving factor for SMEs to adapt, adopt innovations, and enhance business performance. Furthermore, Fahmi et al. (2025), through the lens of knowledge-based theory, argue that current research is still insufficient to explore how digital technology development impacts innovation and business performance among SMEs in the creative economy sector. These findings highlight a research gap regarding the extent to which digital technologies are integrated into business operations, particularly in the context of upcycling, and their role in promoting circular economy practices. This gap underscores the need for empirical studies to clarify how digital adoption translates into operational and strategic value in upcycling craft industry in Indonesia pursuing sustainable and innovative business models.

In this vein, referring to the findings of Fahmi et al. (2025) and Zheng et al. (2025), this study highlights the presence of determinant factors that explain why digital technology adoption remains weak among SMEs. Moreover, Wahmuda et al. (2024) pointed out that most empirical studies focus on the circular economy and sustainability perspectives, whereas empirical research examining digital adoption behavior and innovation transformation in micro-enterprises is almost nonexistent. This underscores a significant research gap: although the upcycling industry has considerable potential for circular innovation, there is still no comprehensive theoretical and empirical framework to explain how digital technologies are integrated into production and innovation processes. Additionally, Prieto-Sandoval et al. (2021) state that, despite the growing attention toward circular economy strategies, structured methodologies for implementing digital innovation and transformation in SMEs—particularly in the creative sector—remain underdeveloped. This perspective aligns with technology adoption theories emphasizing the importance of behavioral dimensions. As proposed by Davis (1989), the Technology Acceptance Model (TAM) asserts that perceived usefulness and perceived ease of use influence user attitudes and behavioral intentions toward technology adoption. Meanwhile, Ajzen (1991) argues in the Theory of Planned Behaviour (TPB) that intention is determined by attitude, subjective norms, and perceived behavioral control, which serve as antecedents of behavior. Additionally, Rogers’ (2003) Innovation Diffusion Theory emphasizes the role of social systems, communication channels, and adopter categories in shaping innovation diffusion. Based on these insights, technology adoption is influenced not only by infrastructure readiness but also by behavioral acceptance, social support, and organizational alignment ( Susanty et al., 2020; Vaska et al., 2021; Venkatesh et al., 2012).

Although TAM, TPB, and IDT provide important conceptual frameworks, they are rarely integrated into a comprehensive model that reflects the complexity of contextual dynamics in micro-scale enterprises. Furthermore, research on upcycling enterprises seldom adopts a behavioral perspective, despite the fact that innovation and technology adoption decisions in this sector are highly personal, influenced by individual skills, and shaped by limited resources. Therefore, this study develops a theoretical framework to explore the extent to which determinant factors in digital technology adoption influence innovation by integrating the behavior of microenterprise owners, particularly in the craft sector of the creative industry. In this area, the craft sector, which transforms waste or discarded products into new, value-added goods, playing a critical role in supporting circular economy principles and sustainable development ( Bridgens et al., 2018; Geissdoerfer et al., 2017; Sung et al., 2019). In addition, the creative industry significantly contributes to Indonesia’s economy, averaging 6% year-on-year, with the craft subsector serving as a major contributor at 14.5% ( KataData Insight, 2024). Based on these considerations, this study formulates three research questions:

RQ1: Which internal and external factors influence digital technology adoption in the upcycling craft industry, and how do industry actors respond to these changes?

RQ2: To what extent is digital technology implemented in the upcycling craft industry, particularly in relation to sustainable production processes and product innovation?

RQ3: What digital transformation model can support the upcycling craft industry in enhancing production efficiency and sustainable product innovation?

## 2. Literature Review

### 2.1 Behavioral Foundations of Digital Transformation in Micro Creative Enterprises

Behavioral foundations represent a critical dimension in explaining why digital transformation succeeds or fails within micro creative enterprises. Classical technology adoption theories—including the Technology Acceptance Model (TAM), the Theory of Planned Behavior (TPB), and the Unified Theory of Acceptance and Use of Technology (UTAUT)—consistently emphasize behavioral intention as a key mechanism linking perceived technology value, social influence, and facilitating conditions to actual technology use ( Davis, 1989; Ajzen, 1991; Venkatesh et al., 2012). Collectively, these frameworks provide a foundational explanation of how individual evaluations and social pressures shape adoption behavior.

When situated within the context of circular and creative economies, behavioral perspectives offer a more nuanced lens for analyzing digital transformation. Sustainability-driven micro-enterprises often operate under significant resource constraints, where behavioral readiness—rather than mere technological availability—becomes a decisive factor enabling digital adoption ( George et al., 2021; Saleh & Brem, 2023). This condition is particularly salient in upcycling craft industries, where entrepreneurs must balance artisanal creativity with the integration of digital tools for marketing, product development, and operational efficiency. The interplay between creative identity, circularity objectives, and technological adaptation introduces behavioral complexities that linear adoption models struggle to capture. Empirical studies further indicate that leadership commitment, innovation culture, and learning orientation enhance digital readiness, whereas skill limitations, financial constraints, and low digital literacy continue to impede adoption progress in micro creative enterprises ( Efendi et al., 2020; Nadkarni & Prügl, 2021).

Despite growing scholarly attention to digital adoption in small firms, existing research tends to treat internal and external factors primarily as contextual or environmental conditions rather than explicitly modeling how they shape behavioral intention as a mediating mechanism. Few empirical studies examine how intention links these determinants to actual digital technology implementation and innovation outcomes, particularly in micro-scale creative and upcycling enterprises. As a result, the behavioral processes through which digital adoption translates into innovation and transformation remain insufficiently understood. Recent studies by Fahmi et al. (2025) and Zheng et al. (2025) highlight that weak digital adoption among micro and small enterprises is often rooted in limited behavioral readiness, including low confidence, fear of failure, and uncertainty regarding the value of digital technologies. Similarly, Wahmuda et al. (2024) observe that research in circular and upcycling contexts has predominantly focused on sustainability outcomes and material recovery, while behavioral dimensions of digital adoption and innovation transformation remain underexplored.

In parallel, Prieto-Sandoval et al. (2021) argue that, despite the widespread promotion of circular economy principles, structured approaches for operationalizing digital innovation in SMEs—particularly within creative sectors—remain underdeveloped. This perspective aligns with behavioral technology adoption theories, which emphasize that successful adoption depends not only on infrastructure readiness but also on behavioral acceptance, social reinforcement, and organizational alignment ( Rogers, 2003; Susanty et al., 2020; Venkatesh et al., 2012). Moreover, arts- and creativity-based assets and processes have increasingly been recognized as catalysts for managerial change, entrepreneurial behavior, business model innovation, and organizational learning (e.g., Schiuma, 2017; Schiuma & Lerro, 2017).

Synthesizing these insights, behavioral intention emerges as a central mechanism linking internal and external enablers with digital technology implementation. Building on this theoretical foundation, the present study proposes the Behavioral-Based Digital Transformation (BBDT) model, positioning behavioral intention as the core driver of digital adoption and innovation outcomes within upcycling craft enterprises. Synthesizing these insights, behavioral intention emerges as a central mechanism linking internal and external enablers with digital technology implementation.

### 2.2 Digital Transformation in Creative Industries

Recent advancements in digital technology have successfully transformed the dynamics of the business environment, which are recognized as fundamental drivers in the competitive landscape ( Amankwah-Amoah et al., 2024; Appio et al., 2021). Over the past decade, the adoption of digital technologies has significantly influenced innovation development and interacted with the field of entrepreneurship, generating industrial, managerial, and policy implications ( Broekhuizen et al., 2021; Casciani et al., 2022; Lerro et al., 2022; Vial, 2019). Firms that adopt digital technologies are reported to occupy a superior position in terms of business models and competitiveness ( Amankwah-Amoah et al., 2024). Accordingly, digital technology serves as a transformative force, synthesizing its effects and impacts on business models, products and services, supply chain configurations, and strategic orientations ( Casciani et al., 2022; Cennamo et al., 2020; Raff et al., 2020). Rather than simply integrating digital technology into business operations, its adoption radically reshapes strategic orientation, thereby determining potential success or failure. As Snowball et al. (2022) suggest, a critical aspect of digital technology adaptation and adoption lies in a firm’s ability to innovate effectively, leveraging technological capabilities to generate and capture value.

Despite the recognition of technology as a key driver in enhancing business agility, knowledge exploitation, and facilitating business model innovation ( Kraus et al., 2021), its effects remain under debate. Many studies have investigated the impact of digital technology primarily in the context of large enterprises (e.g., Li et al., 2023; Liu et al., 2023; Zhai et al., 2022; Zhao et al., 2024). Meanwhile, Lerro et al. (2022) highlighted a broader research gap in studies of digital-based innovation and entrepreneurship, noting that not all industries are equally capable of adopting digital technologies. This aligns with the findings of Fahmi et al. (2025), which emphasize that technology, as a moderating variable, may actually weaken the relationship between innovation adoption and business performance among SMEs in the creative economy sector. This interaction relates to Lerro et al.’s (2022) discussion on what and how to implement and utilize digital infrastructure effectively, leveraging firm knowledge foundations and organizational drivers of technology adoption. Furthermore, Lerro et al. (2022) note that studies providing in-depth attention to the broader Creative and Cultural Industries are still limited.

In this vein, recent literature on digital technology adoption in the creative industry increasingly highlights this need (e.g., Lerro et al., 2022; Snowball et al., 2022). In developing countries, digital transformation has become a crucial factor for SMEs to strengthen competitiveness in global markets ( Crupi et al., 2020; Depaoli et al., 2020). However, many creative SMEs still face challenges related to low digital literacy, limited innovation capacity, and inadequate structural readiness ( Wijaya et al., 2021; Yulianti et al., 2022). These challenges underscore the need for a behavior-based transformation model that integrates organizational, technological, and socio-cultural dimensions in digital adoption. In this context, Silva et al. (2025) proposed a new Lean-based readiness framework that balances technological, organizational, and process capabilities, going beyond traditional approaches that focus solely on technology in digital transformation planning. Silva et al. (2025) emphasize that a comprehensive model must align digital capabilities with internal readiness and an innovation-oriented culture to ensure effective implementation. Meanwhile, a systematic literature review by Wahmuda et al. (2024) revealed that, although digital transformation has been widely explored within the creative and circular economy sectors, research specifically examining the relationship between digitalization and upcycling practices remains extremely limited. Behavioral barriers, resource constraints, and low digital readiness among artisans and micro-entrepreneurs continue to be major obstacles to transformational adoption.

### 2.3 Upcycling and Circular Innovation

Upcycling is widely recognized as a tangible expression of circular economy principles, contributing to reduced resource extraction, extended product life cycles, and minimized environmental impacts ( Geissdoerfer et al., 2017; Korhonen et al., 2018). The circular economy paradigm emphasizes restorative and regenerative production systems designed to retain value, close resource loops, and reduce ecological footprint ( Perotti et al., 2024). Within this framework, upcycling functions as a socially embedded practice that merges ecological consciousness with economic value creation, enabling alternative material pathways and supporting low-carbon production models ( Srisathan et al., 2025). Thus, it embodies an integrated sustainability mechanism that generates shared value through environmental responsibility and creative adaptation. As upcycling is defined as the creative process of transforming discarded materials or waste into higher-value products through design and innovation ( Bridgens et al., 2018; Sung et al., 2019). As a design-led material revalorization strategy, upcycling prioritizes creativity, craftsmanship, and experimentation in redefining the functional and aesthetic worth of previously discarded resources. It represents an evolution beyond conventional recycling, in which materials often undergo degradation in quality through down-cycling processes that limit future usability and value creation ( Bocken et al., 2016; Civera et al., 2025). In contrast, upcycling seeks to elevate material value through artistic reinterpretation and product redesign, reinforcing its role as a catalyst for sustainable and regenerative material flows.

Recent literature highlights the role of digital technologies in enhancing upcycling practices and enabling broader participation in creative recycling systems. Digital tools—including 3D design software, digital fabrication technologies, virtual prototyping, and online collaborative platforms—have been shown to expand design possibilities, accelerate iterative experimentation, and improve product development efficiency ( Aranda-Usón et al., 2020; Chaudhuri et al., 2022; Wijekoon et al., 2025). Participatory design ecosystems supported by digital tools connect artisans, designers, and consumers, fostering collaborative creativity and enabling co-design approaches that strengthen upcycling innovation capacity ( Botero et al., 2020; Sung et al., 2019). Digital ecosystems also transform traditional production logics by enabling transparency, traceability, and storytelling—features that align with consumer demand for ethically driven production and sustainable brands ( Lerro et al., 2022). In developing and emerging economic contexts, digital upcycling plays an additional developmental function by promoting inclusivity, community empowerment, and skills upgrading. Studies by Chappe and Jaramillo (2020) and Wang et al. (2023) demonstrate that digital–craft hybridization enables knowledge transfer, preserves local cultural identity, and strengthens creative entrepreneurship at the community level. The integration of modern digital capabilities with traditional craftsmanship supports livelihood sustainability, increases market reach, and enhances the competitiveness of informal and micro-scale producers, particularly in regions where access to conventional manufacturing infrastructure is limited.

Beyond environmental impact, upcycling contributes to economic resilience and entrepreneurial sustainability within creative industries. Research indicates that SMEs adopting circular business models, including upcycling, experience increased innovation performance, resource efficiency, stakeholder engagement, and competitive differentiation ( Brendzel-Skowera, 2021; Wijekoon et al., 2025). Upcycling fosters value creation through product uniqueness, craftsmanship authenticity, and narrative-driven branding—factors increasingly important in competitive creative markets ( Isaac-Bamgboye et al., 2025). It also stimulates localized economic activity by utilizing locally available waste materials and promoting community-based production networks, reducing dependency on global supply chains and enhancing adaptive capacity during economic disruptions, such as those experienced during the COVID-19 pandemic ( Kraus et al., 2021). Positioned at the intersection of sustainability-oriented entrepreneurship and circular design, upcycling represents both an ecological and entrepreneurial mechanism for sustainable transformation within creative economies. Its integration within the digital transformation agenda expands opportunities for innovation, collaborative production, and market development, especially for micro-enterprises navigating resource scarcity and competitive pressures. Yet, despite its rapidly growing relevance, limited research examines how behavioral readiness and digital capability shape digital upcycling implementation—revealing a critical gap in understanding digital transformation dynamics in sustainability-driven creative sectors.

## 3. Method

This study adopts a mixed methods research design, combining qualitative and quantitative approaches to generate comprehensive insights and overcome the limitations of using a single method ( Greene et al., 1989). Recognized as the “third methodological movement” in research, mixed methods enable the exploration of complex phenomena from multiple perspectives ( Creswell & Clark, 2017; Greene, 2007). Specifically, this study employs an exploratory sequential design, in which qualitative data are collected and analyzed first to gain an in-depth understanding of digital adoption and innovation in upcycling craft enterprises. These initial insights inform the subsequent quantitative phase, guiding the development of survey instruments and measurement constructs tailored to the study context. The final phase involves the quantitative testing of these constructs, allowing the researcher to validate patterns identified in the qualitative phase. By integrating the two stages, the study demonstrates how participants’ experiences and perceptions are expanded or reinforced through quantitative analysis. This approach not only strengthens the robustness of the findings but also provides a holistic view of the behavioral, organizational, and contextual factors influencing digital adoption, implementation, and innovation outcomes in Indonesia’s upcycling craft sector.

**Figure 1.**
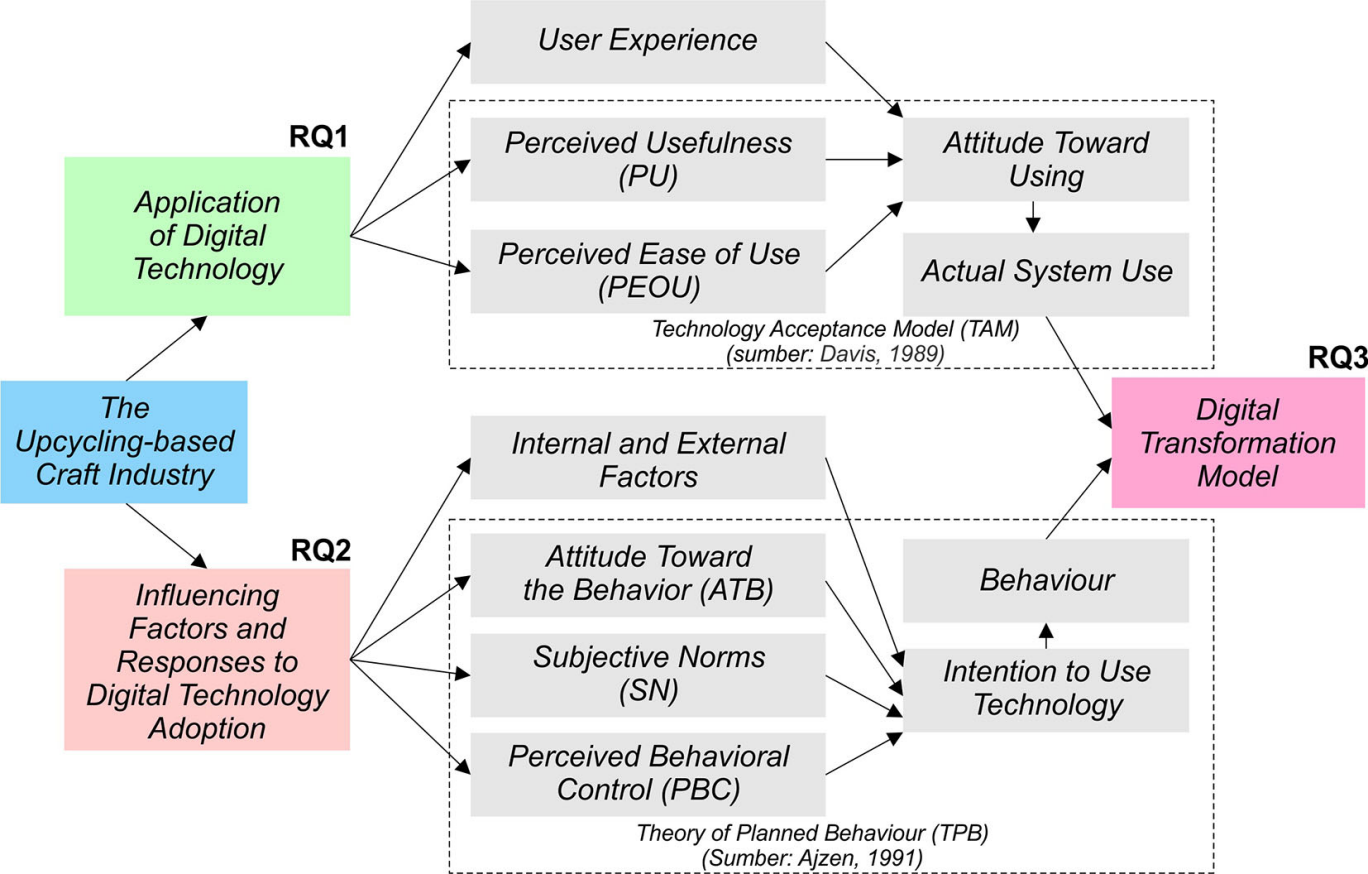
Research Problem-Solving Framework. Figure is an original creation by the authors, specifically designed for this study.

This approach enables an in-depth understanding of digital technology adoption within the upcycling craft industry, while providing exploratory empirical support for the proposed model through quantitative testing. To illustrate the alignment between research questions and methodological stages, the research problem-solving framework is presented in Figure 1 below. This study is underpinned by three key theoretical foundations: the TAM, the TPB, and the IDT. These theories were selected as they complement one another in explaining the processes of digital adoption and transformation from behavioral and innovation perspectives, such as:

(a) The TAM posits that perceived usefulness and perceived ease of use influence an individual’s attitude and intention to use technology ( Davis, 1989).
(b) The TPB extends TAM by incorporating the influence of subjective norms and perceived behavioural control on behavioural intention ( Ajzen, 1991).
(c) The IDT ( Rogers, 2003) explains how digital innovations are adopted and disseminated within organisations and communities.

**Figure 2.**
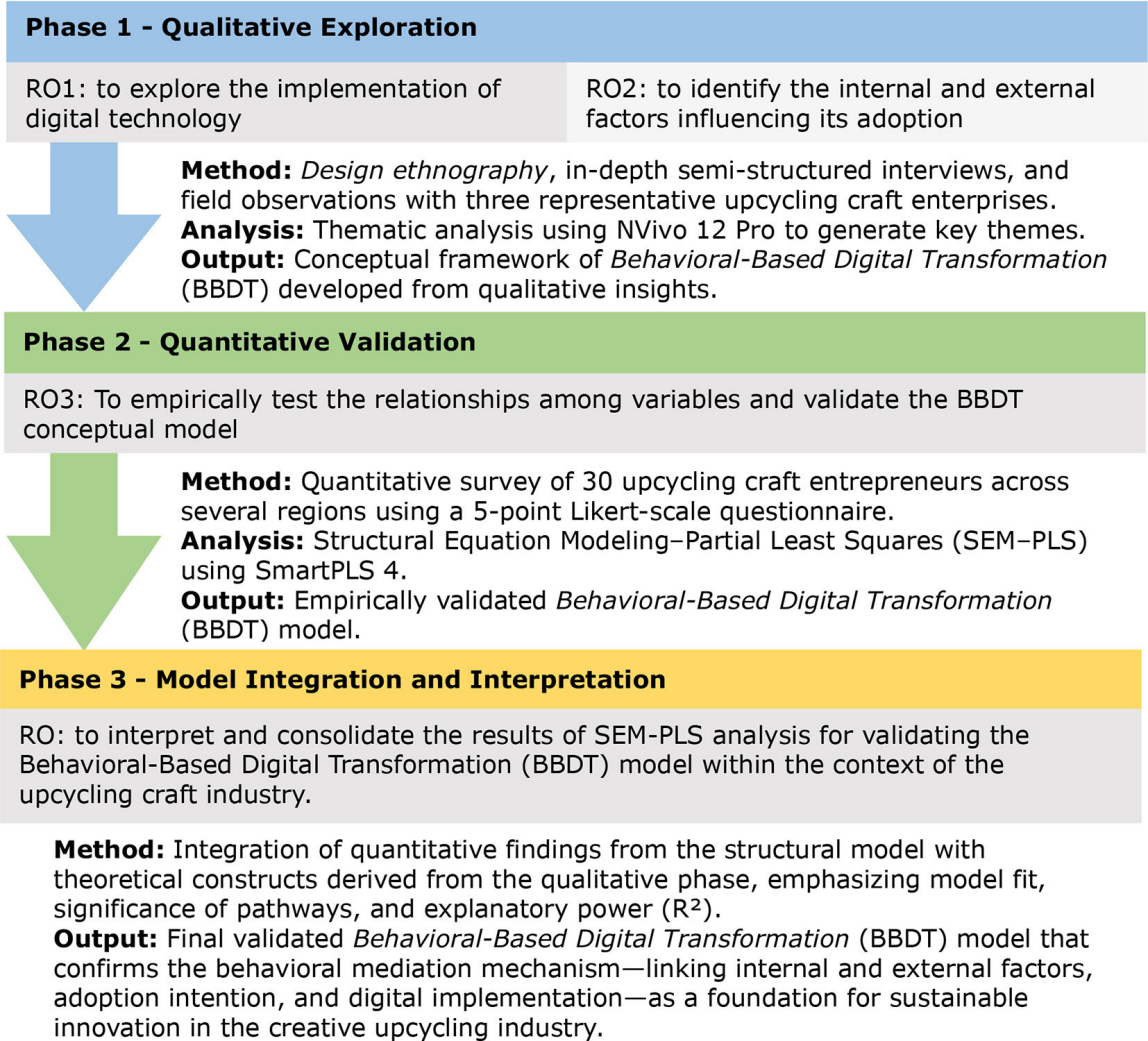
Research Design. Figure is an original creation by the authors, specifically designed for this study.

The combination of these three theoretical perspectives provides the conceptual foundation for the development of the BBDT model, which emphasises that the success of digital transformation in the upcycling craft industry is determined not only by technological readiness but also by behavioural, social, and cultural factors among industry actors. This approach was chosen to gain a comprehensive understanding of the mechanisms of digital technology adoption, the factors influencing it, and how these processes contribute to sustainable product innovation. The stages of the research were systematically designed and are illustrated in Figure 2 below.

### 3.1 A Qualitative Phase

The first phase of the study aims to address RQ1 and RQ2 namely, to explore the implementation of digital technologies and identify the internal and external factors influencing their adoption. The qualitative approach employed a multiple case study of three upcycling craft enterprises in East Java, specifically in Situbondo Regency, Gresik Regency, and Jombang Regency. These cases were selected to represent variations in digitalization levels and types of recycled materials. Data were collected using a design ethnography approach, with active participation in design and production processes to observe techniques, workflows, and the use of digital technologies. In addition to participatory observation, in-depth interviews were conducted with business owners, designers, artisans, and other relevant stakeholders. Interviews lasted 60–90 minutes, were audio-recorded, and transcribed verbatim.

The three cases were selected not only to represent different stages of digital adoption, but also to capture variation in upcycled material characteristics, encompassing both organic and inorganic waste streams, namely seashell waste (MS), corn cobs (KIK), and glass waste for beaded accessories (BF). This material heterogeneity is analytically relevant because different materials impose distinct production constraints, degrees of processing complexity, and levels of compatibility with digital tools such as CNC machining, laser cutting, or digital design software. Consequently, material characteristics influence the feasible scope and pace of digital integration across the upcycling workflow.

Data analysis was performed using thematic analysis with NVivo 12 Pro, following three main steps, including: (1) Open coding – identifying key terms and significant ideas from participant narratives; (2) Axial coding – grouping codes into interrelated thematic categories; and (3) Selective coding – establishing relationships among categories to form initial conceptual constructs. The analysis generated core themes that served as the basis for developing a behavior-based conceptual model of digital transformation in the upcycling craft industry.

### 3.2 A Quantitative Phase

The second phase of the study aims to address RQ3 by examining the relationships among the variables and providing exploratory empirical support for the proposed BBDT model. A survey was conducted involving 30 upcycling craft entrepreneurs across several provinces, including East Java, the Special Region of Yogyakarta, West Nusa Tenggara, Central Java, West Java, North Kalimantan, DKI Jakarta, and other regions in Kalimantan. The selection of upcycling-based craft enterprises refers to data from the National Waste Management Information System (Sistem Informasi Pengelolaan Sampah Nasional—SIPSN), which provides verified information including business names, addresses, and subsector classifications. The questionnaire was developed based on qualitative findings and relevant supporting theories—namely the Technology Acceptance Model (TAM), the Theory of Planned Behavior (TPB), and Innovation Diffusion Theory (IDT)—and was measured using a five-point Likert scale (1 = strongly disagree; 5 = strongly agree).

In the context of owner–operator micro-enterprises, the owner typically acts simultaneously as founder, manager, and primary decision-maker. As a result, attitudinal evaluation and intention formation regarding digital adoption are often enacted by the same individual and within a single decision episode. Prior studies on micro-enterprises and sole proprietorships indicate that owners’ attitudes, aspirations, and personal predispositions directly translate into adoption decisions, making empirical separation between attitude and intention less distinct in practice ( Jones et al., 2014; Vatanasakdakul et al., 2020; Nair et al., 2019). Accordingly, this study operationalizes attitude and intention as a composite construct to capture the owner–operator’s overall digital adoption orientation.

Data analysis was performed using Structural Equation Modelling–Partial Least Squares (SEM–PLS) in SmartPLS 4.0, which is suitable for small sample sizes and models incorporating mediation effects. Hair et al. (2014) assert that PLS-SEM is a robust statistical method because it is applicable to all data scales, imposes fewer assumptions, and can validate relationships even without a strong theoretical basis. PLS-SEM was chosen over other statistical methods due to its distinct advantages in handling complex models, particularly when the research is exploratory in nature or when the sample size is relatively small ( Hair et al., 2014, 2019). PLS-SEM is highly effective in situations where the research model is complex, involving multiple constructs and indicators, and where the goal is to predict key target constructs or identify relationships between variables ( Hair and Alamer, 2022). One of the main reasons for choosing PLS-SEM is its flexibility in handling non-normal data distributions, which can be a limitation in CB-SEM ( Hair et al., 2017, 2022). Unlike CB-SEM, which relies on the assumption of normality, PLS-SEM does not require this assumption, making it more robust in practical research settings where data might not meet these stringent criteria ( Hair et al., 2017, 2022). Additionally, PLS-SEM is advantageous for developing hypotheses, predicting complex scenarios, and conducting multivariate data analysis ( Hair et al., 2019).

Given the limited sample size (n = 30), the application of PLS-SEM in this study should be interpreted as exploratory and predictive hypothesis testing rather than confirmatory analysis. As noted by Hair et al. (2014, 2019), PLS-SEM is particularly suitable for exploratory research and studies with relatively small sample sizes. Accordingly, the statistical findings provide preliminary evidence of the proposed relationships, indicating the need for replication with larger samples to strengthen and complement the qualitative findings through data triangulation. Given the cross-sectional nature of the study, digital transformation is conceptualized as an early-stage behavioral and technological pathway rather than a fully observed longitudinal organizational change. This conceptualization is consistent with prior digital transformation frameworks that distinguish initial readiness or digitization stages from deeper process and business model transformation ( Verhoef et al., 2021; Tian & Ou, 2024; Jia et al., 2024).

### 3.3 Participant and Procedure

The participant selection strategy aligned with the exploratory sequential mixed methods design, in which qualitative participant involvement informs the development of the subsequent quantitative phase. In the qualitative phase, participants were selected through purposive sampling based on criteria related to active involvement in upcycling craft production, the utilization of discarded materials as primary inputs, and engagement with digital technology in at least one business function (e.g., marketing, design, fabrication, inventory management, or customer interaction). Three upcycling craft enterprises located in East Java—specifically in Situbondo Regency, Gresik Regency, and Jombang Regency—were selected due to their diverse levels of digitalization and variation in material types processed. This diversity enabled an in-depth exploration of behavioral, organizational, and technological dynamics across contrasting digital maturity levels. Participants for the qualitative study included business owners, designers, artisans, and relevant institutional partners such as community leaders and craft ecosystem facilitators. Data were collected through participatory design ethnography, in which the researcher was actively embedded in production environments to observe work processes, digital tool usage, collaborative dynamics, and decision-making patterns. Semi-structured interviews lasting 60–90 minutes were conducted with each participant, audio-recorded, transcribed verbatim, and subsequently coded using NVivo 12 Pro.

In the quantitative phase, participants were selected using a criterion-based sampling strategy targeting upcycling craft entrepreneurs operating across multiple Indonesian provinces. The survey instrument consisted of items operationalizing constructs derived from the qualitative thematic findings and theoretical foundations—TAM, TPB, and IDT. In compliance with ethical research standards, the introductory page provided detailed information about the study along with an informed consent statement asking, “Are you willing to participate as a respondent in this study?”. Only respondents who provided consent were allowed to proceed to the subsequent pages. Measures to safeguard data privacy were implemented, including anonymizing responses and securely storing all collected information in accordance with relevant data protection regulations. By emphasizing these ethical considerations, the study adhered to established best practices, ensuring the protection of participants’ privacy and autonomy throughout the research process. Ethical approval was obtained from the institutional research ethics committee, and informed consent was acquired before data collection commenced. To ensure anonymity, identifying information was removed and coded prior to analysis.

### 3.4 Ethical Approval

This research has obtained ethical approval from the Ethics Committee of Interdisciplinary School of Management Technology, Institut Teknologi Sepuluh Nopember, Indonesia. The study was reviewed to ensure compliance with ethical standards regarding research involving human participants, including informed consent, confidentiality protection, and voluntary participation procedures. The protocol of this study was evaluated and approved under the reference number 9839/IT2.IX.8/B/TU.00.09/XI/2025. All participants were informed about the objectives and processes of the study and provided written informed consent prior to participation. Respondents were assured that their participation was voluntary, that they could withdraw at any time without consequences, and that their identity and responses would remain anonymous. No personal identifying information was collected, and the data were used solely for academic research purposes. The study strictly adhered to the ethical principles outlined in the Declaration of Helsinki (2013) and the General Data Protection Regulation (GDPR) standards for data privacy.

## 4. Result and Analysis

### 4.1 Qualitative Phase Results: Exploration of Digital Technology Adoption and Influencing Factors

The qualitative phase aimed to explore how digital technology is adopted within the upcycling craft industry and to identify both internal and external factors influencing the adoption process. The analysis was conducted using field observations and in-depth interviews with three upcycling craft enterprises located in Situbondo Regency, Gresik Regency, and Jombang Regency. Table 1 below presents the demographic profile of participants in the qualitative phase.

A. **Case Overview: Digital Adoption in Three Upcycling Craft Enterprises**

**Table 1.** The demographic profile of participants in the qualitative phase.

Key informant	Gender	Position	Business age	Number of employee	Product material	Place of origin	Code
MS	Male	Owner	23 years	10 employees	Seashell waste – Interior decoration	Situbondo Regency	MS
BF	Male	Owner	35 years	23 employees	Glass waste – Beaded accessories	Jombang Regency	BF
KIK	Male	Founder and Designer	7 years	6 employees	Corn cobs – Interior decoration	Gresik Regency	KIK

**Table 2.** Summarizes the three enterprises (MS, KIK, BF) against key themes, highlighting patterns and differences. Source: Authors’ analysis based on interview data ( Wahmuda et al., 2025).

Cross-Case Synthesis								
Case	Digital adoption stage	User experience	Perceived usefulness	Perceived ease of use	Internal factors	External factors	Behavioral intentions	Key observations
MS	Advanced	Positive, high mastery	High – efficiency & quality	High – confident operators	Strong leadership, innovation culture	Customer demand	Strong intention to sustain	Full integration in core production processes
KIK	Transitional	Moderate – partial mastery	Moderate – supports prototyping	Moderate – limited operator training	Adequate leadership, moderate resources	Customer demand	Moderate intention	Limited integration, relies on partial digital tools
BF	Early	Low – hesitant	Low – basic marketing & visualization	Low – digital literacy limited	Limited resources, training	Market demand primary	Weak intention	Early adoption stage, high reliance on manual work

MS represents an advanced adopter within the upcycling craft industry, having successfully integrated CNC machinery and laser engraving into its production workflow. The enterprise employs digital tools to optimize seashell waste, resulting in high design precision and consistent product quality. The inorganic and rigid characteristics of seashell waste provide material stability that is highly compatible with subtractive digital fabrication technologies, such as CNC machining and laser engraving, enabling precise cutting, engraving, and repeatable pattern production. The adoption of digital fabrication technologies has not only reduced production time and material waste but has also enabled creative experimentation with complex patterns. This digital integration has strengthened operational efficiency while cultivating a culture of innovation within the workshop, where artisans combine traditional handcrafting techniques with computer-aided design to create unique, high-value, and sustainable products.

KIK demonstrates a transitional stage of digital adoption, characterized by the partial integration of 3D design software and laser cutting technology. The enterprise uses digital design tools for product prototyping, enhancing accuracy in cutting recycled materials and supporting mass customization. As an organic and heterogeneous material, corn cobs present higher variability in texture, density, and durability, which requires iterative prototyping and limits the full automation of digital fabrication processes. Digitalization has improved material efficiency and facilitated diversification of product lines—particularly in furniture and decorative crafts—allowing the company to penetrate new market segments. However, the adoption process remains limited by the availability of trained digital operators and access to affordable technological upgrades. This indicates that innovation readiness within small creative enterprises requires capacity building in both technical and human resource dimensions.

BF represents an early-stage adopter, focusing primarily on the initial utilization of digital design and online marketing tools rather than full-scale production digitalization. The enterprise employs basic software applications for product visualization and relies heavily on social media platforms to expand market access. Although the entrepreneurial actor acknowledges the potential of digital tools to enhance production efficiency and business visibility, BF faces significant constraints related to digital literacy, investment in equipment, and availability of skilled labor. The owner’s proactive attitude reflects a strong behavioral intention toward digital transformation; however, resource limitations and insufficient institutional support hinder the realization of adoption goals. This case exemplifies the behavioral and structural challenges faced by micro-scale upcycling enterprises in the early stages of digital adoption.

B. **Thematic Analysis of Digital Adoption in the Upcycling Craft Industry**

The qualitative exploration identified five main themes: User Experience, Perceived Usefulness, Perceived Ease of Use, Internal and External Factors, and Behavioral Intentions. Collectively, these themes capture the behavioral and contextual dynamics underlying digital adoption within the upcycling craft industry. The themes were synthesized from interview transcripts and observational data using NVivo 12 Pro and aligned with the constructs of the Technology Acceptance Model (TAM) and the Theory of Planned Behavior (TPB), such as:

(1) User Experience reflects artisans’ initial interactions with digital tools such as CNC machines, laser engraving technology, and 3D design software. Participants commonly expressed dual perceptions in which digital tools were initially experienced as complex and intimidating, yet ultimately regarded as highly rewarding once mastered. This finding aligns with the TPB construct of attitude toward behavior, indicating that hands-on engagement shapes positive perceptions of technology. Artisans from MS and KIK reported that digital tools enhanced design precision and production consistency, thereby increasing creative confidence and operational efficiency.
(2) Perceived Usefulness emerged as the dominant factor influencing digital adoption behavior. Respondents repeatedly emphasized that digital technologies increased productivity, improved product quality, and reduced material waste—key motivations for integration into production systems. This supports the TAM principle that perceived usefulness directly affects behavioral intention. For example, the implementation of CNC machinery at MS reduced production time by half, while KIK’s adoption of laser cutting enabled diversification into new product lines, illustrating how perceived utility translates into tangible operational outcomes.
(3) Perceived Ease of Use moderated artisans’ willingness to adopt and sustain digital technology practices. Although participants acknowledged the substantial benefits, many—particularly from BF—expressed reluctance due to limited digital literacy and inadequate structured training. This indicates that technological availability alone is insufficient without comprehensive skills development. Challenges in mastering design software such as CorelDRAW and AutoCAD frequently resulted in dependence on external operators, reducing artisans’ autonomy in managing digital processes.
(4) Internal and External Factors explain the structural and environmental conditions influencing adoption decisions. Internally, leadership openness to innovation, financial capacity, and organizational culture were identified as critical enablers. Externally, market demand and customer expectations emerged as stronger motivators than government policies or institutional support programs. This aligns with the TPB construct of subjective norms, demonstrating that social and market pressures play a more significant role in adoption behavior than regulatory frameworks. Cross-case synthesis revealed that enterprises responding to customer-driven customization demands integrated digital technologies more rapidly.
(5) Behavioral Intentions function as a mediating mechanism linking Perceived Usefulness and Perceived Ease of Use with actual technology implementation. Artisans with positive attitudes and high self-efficacy demonstrated stronger intentions to continuously invest in and learn digital tools. Participants described digital transformation as an ongoing learning journey, reflecting the gradual innovation diffusion process outlined in Rogers’ Innovation Diffusion Theory (IDT) ( Rogers, 2003). These intentions were shaped not only by perceived technological benefits but also by community support and collaborative peer learning among artisans.

Thus, the thematic analysis demonstrates a coherent behavioral pattern consistent with TAM and TPB frameworks: Perceived Usefulness and Perceived Ease of Use shape Attitudes and Behavioral Intentions, moderated by Internal and External Factors. These behavioral drivers underpin the early phases of digital transformation in the upcycling craft industry, highlighting that technological readiness alone is insufficient without parallel development of human capability and organizational support systems.

C. **NVivo Analysis: Key Findings from Cross-Case Coding**

The NVivo-based cross-case analysis revealed consistent behavioral and contextual patterns across the three case studies. Although digital technologies—such as CNC machining, laser cutting, and digital design software—had been introduced in all enterprises, their utilization remained partial and not yet fully optimized. Most digital tools were applied only at specific stages of production (e.g., cutting or engraving), while design ideation and finishing processes continued to rely heavily on manual craftsmanship. This suggests that digital transformation within the upcycling craft industry is still in an early integration phase, where digital and traditional practices coexist. A key insight from the analysis concerns the primary barriers to digital adoption, which were predominantly related to technical skills and organizational readiness rather than financial constraints. Artisans frequently lacked structured digital training, resulting in low confidence in operating new equipment or software. Consequently, many enterprises depended on external operators for digitally enabled tasks, restricting knowledge transfer and limiting the potential for sustained digital integration. This underscores the crucial role of digital literacy development in supporting transformation among micro and small creative enterprises.

Furthermore, market demand emerged as a stronger driver than governmental or institutional support. Decisions to adopt digital tools were primarily influenced by customer preferences for precision, customization, and environmentally responsible products. In contrast, policy incentives and government programs were perceived as less accessible or misaligned with the specific needs of small-scale artisans. This finding reinforces the TPB construct of subjective norms, wherein social and market pressures exert a greater influence on behavioral intention than regulatory encouragement. Finally, innovation was found to act as a behavioral enabler, mediating the relationship between technological readiness and adoption intention. Enterprises that engaged in continuous experimentation—such as developing new product variations or combining traditional and digital materials—demonstrated stronger commitment to sustaining digital transformation. In this regard, innovation served not merely as an outcome of technology use, but as a catalyst that reinforced artisans’ positive attitudes and long-term motivation toward digital integration.

D. **Interpretation and Conceptual Model of Digital Transformation**

The qualitative findings indicate that the digital transformation process within the upcycling craft industry follows a behavioral pathway driven by the interaction of internal and external factors, attitudes and intentions, digital adoption, and innovation. Internal factors, such as leadership commitment, financial capability, and an innovation-oriented culture, form the foundation for technology adoption, enabling artisans to perceive digitalization not as a disruption but as an opportunity for creative enhancement. Meanwhile, external factors—particularly market dynamics and customer expectations—act as reinforcing pressures that shape artisans’ perceived need to integrate digital tools into their production and design processes. These dual influences align with the TPB construct of subjective norms and the TAM concept of external variables, both of which precede the formation of behavioral intention toward technology use. Positive attitudes and intentions emerge as the central mediating mechanism linking enabling factors with actual digital implementation. Artisans who perceive technology as useful and manageable tend to exhibit a stronger commitment to adopting and sustaining digital practices. This finding is consistent with the TAM premise that perceived usefulness and perceived ease of use directly shape behavioral intention. However, the qualitative evidence also highlights that, in micro-scale industries, this process is gradual and learning-oriented—reflecting a progressive evolution from awareness to acceptance and, ultimately, to routine integration. Behavioral readiness, rather than infrastructure readiness, thus becomes the primary determinant of successful digital adoption in the upcycling craft context.

Ultimately, digital adoption fosters innovation, serving both as an outcome and as an enabler of transformation. Once artisans engage with digital tools, new creative possibilities emerge—ranging from design customization and material optimization to product diversification. Innovation, in turn, reinforces motivation for continuous technological improvement, creating a cyclical relationship between digital use and creative development. This iterative process represents the early stage of a broader transformation trajectory, in which digitalization becomes embedded not merely as a technical change but as a cultural shift within the creative ecosystem. The resulting BBDT model conceptualizes transformation as a human-centered, early-stage evolutionary process internal and external enablers shape attitudes and intentions, which drive digital adoption, ultimately leading to sustainable innovation and transformation-relevant mechanisms within the organization.

**Figure 3.**
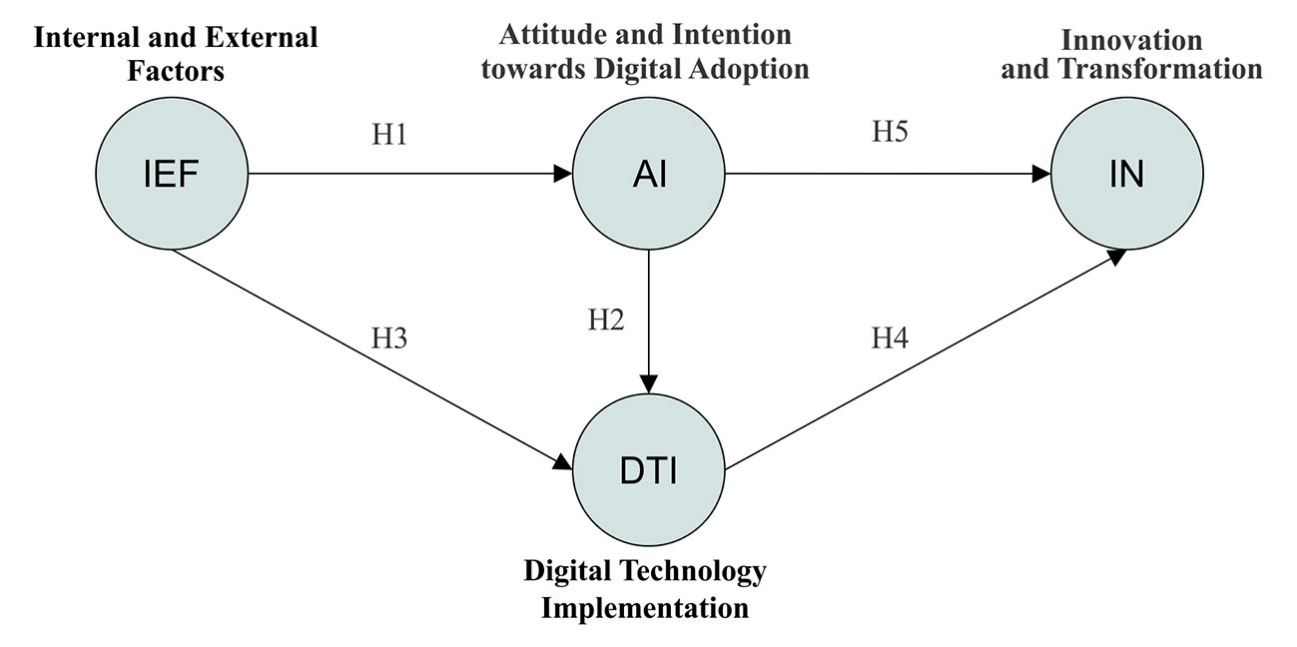
Behavioral-Based Digital Transformation (BBDT) Conceptual Model. Figure is an original creation by the authors, specifically designed for this study.

Figure 3 illustrates the proposed BBDT conceptual model, synthesizing the qualitative findings into a coherent conceptual framework. The model depicts how internal and external enablers influence attitudes and behavioral intentions, which in turn drive digital adoption and foster sustainable innovation within upcycling craft enterprises. Based on the TAM, the TPB, and the IDT, this research model was developed to explain the relationships between IEF, AI, DTI, and IN within the context of the upcycling craft industry.

### 4.2 Quantitative Phase Results: Behavioral-Based Digital Transformation Model Testing

The quantitative phase of this study involved 30 participants from the upcycling-based craft industry, distributed across various provinces, including East Java, Central Java, West Java, the Special Region of Yogyakarta, West Nusa Tenggara, North Kalimantan, DKI Jakarta, and other regions in Kalimantan. Based on Table 3, it presents the demographics of respondents, with a slightly higher proportion of males (58%) than females (42%). The majority of respondents were aged between 35 and 44 years (40%), followed by those aged 45–54 years (33%), 25–34 years (20%), and 55 years or older (7%). In terms of position, most participants were business owners (80%), with a smaller proportion serving as managers (13%) or designers/technical staff (7%). Regarding educational background, over half of the respondents held a bachelor’s degree (53%), followed by senior high school graduates (30%) and master’s degree holders (17%). In terms of upcycling product categories, respondents were primarily involved in fashion and accessories (30%), followed by household and decorative products (27%), furniture and interior products (20%), artistic and craft products (13%), and functional and innovative products (10%). These demographics reflect a diverse group of micro and small-scale entrepreneurs engaged in various segments of the Indonesian upcycling craft industry.

**Table 3.** Demography of Respondents.

Demographic		Frequency	Percentage (%)
Gender	Male	16	58.18
	Female	14	41.82
Age	25–34 years	6	20
	35–44 years	12	40
	45–54 years	10	33
	≥55 years	2	7
Position	Owner	24	80
	Manager	4	13
	Designer	2	7
Education	Senior high school	9	30
	Bachelor’s degree	16	53
	Master’s degree	5	17
Upcycling Product Category	Fashion & accessories products	9	30
	Household & decorative products	8	27
	Furniture & interior products	6	20
	Artistic & craft products	4	13
	Functional & innovative products	3	10

PLS-SEM was employed to assess the validity and reliability of the measurements, serving as the foundation for the quantitative approach (see Table 4). The measurement model (outer model) evaluation was conducted to assess the validity and reliability levels of the constructs within the BBDT model. The assessment comprised three main stages: convergent validity, discriminant validity, and construct reliability ( Hair et al., 2017). Convergent validity was examined based on the values of outer loadings and the Average Variance Extracted (AVE). The results indicated that all indicators had loading factors above 0.70 and AVE values exceeding 0.50, suggesting that each indicator appropriately represented its respective construct ( Hair et al., 2014). Hence, all latent variables were confirmed to meet the criteria for convergent validity. In addition, PLS-SEM evaluates reliability through composite reliability and Cronbach’s alpha, both of which are critical for confirming measurement consistency and must also surpass the 0.70 threshold ( Hair et al., 2019). The results of the measurement analysis in this study indicate that all items and constructs meet these criteria, with values above 0.70. Meanwhile, Discriminant validity was assessed using the Heterotrait–Monotrait Ratio (HTMT). According to the guideline proposed by Henseler et al. (2015), discriminant validity is considered satisfactory when HTMT values are below 0.90. All HTMT values in this research were found to be below the 0.90 benchmark, indicating that the constructs are sufficiently distinct and do not display problematic levels of conceptual overlap. These findings confirm that each construct is distinct and that multicollinearity among variables is not a concern. The outer model evaluation results demonstrate that all constructs in the BBDT model satisfy the measurement adequacy criteria. Therefore, the model is deemed valid and reliable for subsequent structural analysis.

**Table 4.** Construct Measurement.

Variable	Composite Reliability	Cronbach’s Alpha	AVE
Internal and External Factors	0.91	0.87	0.71
Attitude and Intention toward Digital Adoption	0.89	0.85	0.68
Digital Technology Implementation	0.87	0.82	0.64
Innovation and Transformation	0.91	0.88	0.77

The structural model (inner model) evaluation was conducted to examine the causal relationships among the latent variables within the BBDT model. The analysis employed the bootstrapping technique with 5,000 resamples to obtain significance values (t-statistics and p-values), as recommended by Hair et al. (2014, 2017). Additionally, during the bootstrapping stage in PLS-SEM, model fit and path coefficients were analyzed to assess the overall relationships within the model and to test the proposed hypotheses. The application of a partial sequential model in the statistical analysis produced the coefficient of determination (R ^2^), which indicates the explanatory power of the model. R ^2^ is commonly used to evaluate the strength of endogenous constructs within structural models, providing insights into their predictive relevance ( Hair et al., 2022). In the present study, the R ^2^ values were 0.804 for Attitude and Intention toward Digital Adoption, 0.881 for Digital Technology Implementation, and 0.551 for Innovation and Transformation.

As presented in Table 5, the study tested the hypotheses using the bootstrapping procedure in PLS-SEM. The findings indicate that Internal and External Factors had a positive and significant effect on Attitude and Intention toward Digital Adoption (β = 0.902; p < 0.05), supporting H1. Furthermore, Attitude and Intention toward Digital Adoption positively and significantly influenced Digital Technology Implementation (β = 0.894; p < 0.05), supporting H2. However, Internal and External Factors had a positive but non-significant effect on Digital Technology Implementation (β = 0.045; p > 0.05), leading to the rejection of H3. Similarly, Digital Technology Implementation had a positive but non-significant effect on Innovation and Transformation (β = 0.147; p > 0.05), resulting in the rejection of H4. In contrast, Attitude and Intention toward Digital Adoption positively and significantly affected Innovation and Transformation (β = 0.737; p < 0.05), supporting H5.

**Table 5.** Statistical Effect and Hypotheses Testing.

Hypotheses		Direct effect (β)	Indirect effect (β)	T Score	P Values *	Conclusion
H1	IEF ➔ AI	0.902		40.385	0.000	Accepted
H2	AI ➔ DTI	0.894		6.889	0.000	Accepted
H3	IEF ➔ DTI	0.045		0.313	0.754	Rejected
H4	DTI ➔ IN	0.147		0.429	0.668	Rejected
H5	AI ➔ IN	0.737		10.900	0.000	Accepted
H6	IEF ➔ AI ➔ DTI		0.807	7.125	0.000	Accepted
H7	IEF ➔ AI ➔ IN		0.665	9.841	0.000	Accepted

* Sig. p-value < 0.05.

Note: IEF: Internal and External Factors; AI: Attitude and Intention toward Digital Adoption; DTI: Digital Technology Implementation; IN: Innovation and Transformation.

The study also investigated mediation effects. Attitude and Intention toward Digital Adoption positively and significantly mediated the relationship between Internal and External Factors and Digital Technology Implementation (β = 0.807; p < 0.05), confirming H6 as full mediation. Similarly, Attitude and Intention toward Digital Adoption positively and significantly mediated the relationship between Internal and External Factors and Innovation and Transformation (β = 0.665; p < 0.05), confirming H7 as a significant mediation.

Given the exploratory nature of the analysis and the limited sample size, the statistical relationships identified in this study should be interpreted as indicative patterns rather than definitive causal effects. In particular, the strong mediating effects observed for the combined Attitude and Intention construct reflect overall behavioral readiness toward digital adoption rather than distinct attitudinal or intentional mechanisms. Accordingly, the observed relationships should be interpreted as capturing early-stage digital adoption outcomes and transformation-relevant behaviors, rather than mature or fully institutionalized digital transformation. This interpretation aligns with multi-stage digital transformation models, which characterize initial phases as focused on readiness formation, limited implementation depth, and exploratory integration of digital tools ( Berghaus & Back, 2017; Verhoef et al., 2021; Tian & Ou, 2024).

## 5. Discussion and Implication

### 5.1 Discussion

The findings of this study provide compelling insights into the behavioral dynamics, organizational conditions, and digital technology adoption patterns within the upcycling craft industry, highlighting both facilitators and barriers in micro-scale creative enterprises. This research specifically aims to address the significant research gap identified by Lerro et al. (2022), which highlights the limited understanding of how micro-scale upcycling craft businesses in the creative sector implement digital technologies to support innovation and early-stage sustainable transformation pathways. By exploring both internal and external enablers, attitudes, and behavioral intentions, this study investigates how digital adoption translates into innovation outcomes in the upcycling craft industry. Consistent with prior research on digital transformation and small enterprise innovation ( Matt et al., 2015; Vial, 2019), the study demonstrates that internal and external enablers significantly shape artisans’ attitudes and behavioral intentions toward technology adoption. Internal factors, such as leadership commitment, financial capacity, and an innovation-oriented culture, emerged as critical determinants that enable artisans to perceive digital tools as opportunities rather than disruptions. These findings align with the Technology Acceptance Model (TAM) and the Theory of Planned Behavior (TPB), which posit that attitudes toward technology are strongly influenced by perceived usefulness and perceived ease of use ( Ajzen, 1991; Davis, 1989).

The case of MS, an advanced adopter, illustrates how a robust combination of technical readiness and behavioral intention can facilitate advanced levels of digital integration within production workflows such as CNC machining and laser engraving into production workflows. This mirrors findings from the literature suggesting that proactive leadership and innovation culture are critical enablers of technology adoption and operational efficiency in creative enterprises ( Susanty et al., 2020; Vaska et al., 2021). By contrast, KIK and BF represent transitional and early-stage adoption, respectively, revealing the complex interplay between perceived benefits, technological skills, and organizational support. Specifically, KIK’s partial adoption demonstrates that even when perceived usefulness is high, limitations in operator training and digital literacy can constrain full utilization of digital tools ( Ivančić et al., 2019; Nylén & Holmström, 2015). Similarly, BF highlights the challenges micro-enterprises face when structural and financial resources are limited, despite strong behavioral intentions. These results support prior studies emphasizing that SMEs often face capacity and skill constraints that impede digital adoption ( Chaudhuri et al., 2022; Fahmi et al., 2025).

The thematic analysis further underscores the central role of behavioral intention as a mediator between enabling factors and technology implementation. Aligning with Rogers’ Diffusion of Innovation Theory ( Rogers, 2003), artisans’ engagement with digital tools is gradual and experiential, with early positive experiences. Reinforcing self-efficacy and motivation for continued adoption at early stages. Unlike prior TAM–TPB studies predominantly conducted in larger SMEs or formal organizational settings, the findings of this study suggest that in micro-scale upcycling enterprises behavioral intention is closely tied to immediate production experiences and material outcomes rather than abstract performance expectations. Perceived usefulness and ease of use are not evaluated separately, but emerge through hands-on experimentation, iterative learning, and direct engagement with digital tools in everyday production practices.

Interestingly, external pressures, particularly customer demand for high-quality, sustainable, and customizable products, were more influential than government policies or institutional programs, reflecting the TPB notion of subjective norms where social and market pressures outweigh formal regulations ( Ajzen, 1991; Snowball et al., 2022). Enterprises responding to market-driven customization integrated digital tools more rapidly, highlighting the strategic role of customer-oriented innovation in micro-scale creative industries ( Hassoun et al., 2022; Srisathan et al., 2025). This finding aligns with prior studies emphasizing that micro-scale upcycling craft businesses in creative sectors often rely on market feedback to guide technological adoption and innovation ( Geissdoerfer et al., 2017; Singh, 2022).

The quantitative results provide further empirical support for the Behavioral-Based Digital Transformation (BBDT) model, showing strong predictive relationships. The significant mediation effect of Attitude and Intention toward Digital Adoption reinforces the critical role of behavioral readiness ( Centobelli et al., 2020; Civera et al., 2025; Perotti et al., 2024). Conversely, the non-significant effect of internal and external factors on Digital Technology Implementation highlights that structural resources alone are insufficient without behavioral and cognitive alignment, corroborating findings by Lerro et al. (2022) and Fahmi et al. (2025). Furthermore, the mediation of behavioral intention in linking enablers to innovation and transformation supports the concept that digital adoption not only facilitates operational improvements but also catalyzes sustainable innovation within creative micro-enterprises ( Bridgens et al., 2018; Schiuma & Lerro, 2017).

While TAM and TPB conceptually distinguish between attitude and intention, empirical studies in micro-enterprises and MSMEs consistently show that attitude strongly and directly shapes intention, particularly when decision-making authority is highly centralized in the owner–operator. In such contexts, attitude and intention tend to operate as an integrated behavioral orientation rather than as sequentially separable constructs ( Vatanasakdakul et al., 2020; Jones et al., 2014; Rahmawaty et al., 2025; Adu et al., 2022). The present findings align with this stream of research, suggesting that behavioral readiness in micro-scale upcycling enterprises reflects a unified cognitive–motivational stance toward digital adoption.

Importantly, the findings should be situated within the broader literature on early-stage digital transformation. Prior studies consistently distinguish initial phases of digital transformation—often labeled as digitization, initial readiness, or fuzzy front-end stages—from deeper organizational and business model transformation ( Berghaus & Back, 2017; Leipzig et al., 2017; Verhoef et al., 2021). In these early phases, organizations typically focus on exploratory adoption, foundational capability building, and the formation of digital orientation rather than on fully integrated transformation outcomes.

The present study aligns with this perspective by capturing how behavioral readiness, attitudes, and intentions shape the initiation and early implementation of digital technologies in micro-scale upcycling enterprises. Similar to findings in SMEs and other resource-constrained organizations, external pressures and perceived usability tend to dominate early-stage adoption, while organizational structures and advanced digital capabilities become more influential in later stages ( Jia et al., 2024; Zhang et al., 2025). Thus, the BBDT model should be interpreted as explaining early-stage behavioral pathways that enable digital transformation to begin, rather than as a model of mature or completed transformation.

Overall, these findings emphasize a nuanced, behaviorally driven early-stage pathway of digital transformation in upcycling craft enterprises. The iterative cycle of perceived usefulness, skill development, and continuous experimentation fosters innovation, indicating that digital technologies function both as enablers and outcomes of creative practices at the initial stages of adoption ( Schulte et al., 2025). By integrating TAM, TPB, and IDT, this study contributes a behaviorally grounded framework that captures how cognitive readiness, organizational conditions, and market dynamics jointly shape early digital adoption processes in micro-scale creative enterprises. The findings further suggest that capacity-building initiatives, peer learning networks, and customer-driven experimentation are particularly critical for supporting early-stage digital transformation within the context of upcycling craft micro-enterprises ( Cennamo et al., 2020; Venkatesh et al., 2012).

### 5.2 Theoretical Implication

The findings of this study offer several theoretical implications for understanding digital transformation and innovation within micro and small upcycling craft enterprises in Indonesia. First, the study contributes to the integration of multiple theoretical perspectives, including the Technology Acceptance Model (TAM), Theory of Planned Behavior (TPB), and Innovation Diffusion Theory (IDT), into a coherent framework. By synthesizing these models, the Behavioral-Based Digital Transformation (BBDT) framework demonstrates how internal and external enablers, attitudes, and behavioral intentions collectively influence digital adoption and innovation outcomes. This approach addresses a notable gap in the literature identified by Lerro et al. (2022), who emphasized the lack of comprehensive frameworks explaining the digitalization process in micro-scale creative enterprises. Second, the study highlights the pivotal role of behavioral intention as a mediating mechanism linking perceived usefulness, perceived ease of use, and structural enablers to actual digital technology implementation. This finding extends the TAM and TPB literature by illustrating that in micro-scale upcycling craft businesses, technology adoption is not solely dependent on infrastructure readiness but is also profoundly shaped by individual attitudes, self-efficacy, and social pressures ( Susanty et al., 2020; Venkatesh et al., 2012). Third, the research underscores the dynamic interplay between digital adoption and innovation, showing that digital tools act not only as enablers but also as catalysts for continuous creative experimentation and sustainable product development. This insight enriches the IDT literature by demonstrating that innovation in micro-scale craft enterprises emerges through iterative engagement with digital technologies, reinforcing the importance of behavioral and organizational readiness in driving transformative outcomes ( Civera et al., 2025; Rogers, 2003).

### 5.3 Practical Implication

Given that this study captures digital adoption at an early stage, the practical implications should be interpreted as guidance for initiating and strengthening digital transformation pathways rather than prescribing long-term outcomes. The findings of this study have several practical implications for key stakeholders in the upcycling craft industry. For policymakers, particularly the Ministry of Creative Economy and regional government agencies, the results highlight the importance of designing targeted support programs that enhance digital literacy, provide accessible technological resources, and foster an innovation-oriented ecosystem for micro and small upcycling craft enterprises in Indonesia. By prioritizing interventions that address behavioral and organizational readiness, policymakers can facilitate more effective adoption of digital technologies, ultimately promoting sustainable practices within the upcycling sector. For creative economy communities at both national and regional levels, the findings underscore the value of peer learning networks, collaborative workshops, and knowledge-sharing platforms. These initiatives can strengthen artisans’ confidence in using digital tools, encourage experimentation with new materials and designs, and stimulate collective innovation, supporting both competitiveness and circular economy objectives. In addition, educational and research institutions can leverage the insights from this study to develop curriculum and training programs that integrate technical skills with behavioral and managerial competencies. By aligning academic programs with industry needs, these institutions can prepare a workforce capable of driving digital transformation and sustainable innovation within the creative sector. Finally, owners and managers of upcycling craft enterprises can benefit from understanding the critical interplay between internal enablers, attitudes, and behavioral intentions in digital adoption. By strategically investing in digital tools, upskilling personnel, and fostering an innovation-oriented culture, entrepreneurs can enhance operational efficiency, expand market reach, and achieve sustainable growth in an increasingly competitive creative economy.

## 6. Conclusion, Limitation, and Future Research

This study aimed to investigate the adoption of digital technology within the upcycling craft industry, addressing the research gap identified by Lerro et al. (2022) regarding limited empirical evidence on digital adoption and innovation behavior in micro-scale creative enterprises. Specifically, the research sought to examine the internal and external factors influencing digital adoption, the extent of technology implementation, and the resulting impact on innovation and transformation. By integrating the Technology Acceptance Model (TAM), the Theory of Planned Behavior (TPB), and Innovation Diffusion Theory (IDT), this study developed the Behavioral-Based Digital Transformation (BBDT) model to explain the relationships between enabling factors, behavioral intentions, digital technology implementation, and innovation outcomes in upcycling craft enterprises.

The qualitative findings revealed that digital adoption follows a behavioral pathway mediated by attitudes and intentions, shaped by internal factors such as leadership, financial capacity, and innovation-oriented culture, as well as external factors including market demand and customer expectations. Artisans with positive attitudes and higher self-efficacy were more likely to engage in digital practices, combining traditional craftsmanship with digital tools to achieve product diversification, precision, and sustainability. The quantitative analysis indicated that internal and external factors influenced attitudes and intentions toward digital adoption, which in turn were associated with digital technology implementation and innovation-related outcomes. Behavioral intentions also functioned as a key mediating mechanism, highlighting the central role of human behavior in shaping early-stage digital transformation pathways within the upcycling sector. These implications should be interpreted within the context of Indonesian upcycling craft micro-enterprises, where resource constraints, informal work structures, and owner-centered decision-making may differ from larger SMEs or other creative sectors.

Despite these contributions, this study has limitations. The sample size in the quantitative phase was relatively small and limited to select regions in Indonesia, which may constrain the generalizability of the findings. Accordingly, the quantitative findings are limited to providing exploratory insights rather than confirmatory evidence and primarily function to complement the triangulation of the qualitative findings. Consequently, the quantitative results cannot yet be fully generalized, and the application of a purely quantitative approach may yield different findings. In addition, the cross-sectional design limits the ability to observe progression across digital transformation stages. Consistent with multi-stage digital transformation frameworks, future research should adopt longitudinal designs to examine how early-stage behavioral readiness and initial digital adoption evolve into deeper process integration and organizational transformation over time ( Verhoef et al., 2021; Tian & Ou, 2024). Another limitation concerns the aggregation of attitude and intention into a single construct. While this operationalization is contextually justified for owner-managed micro-enterprises, it limits analytical granularity and may obscure potential sequential effects between attitude formation and intention enactment. Future studies should disentangle these constructs to examine differential behavioral pathways using larger samples and longitudinal designs.

Building on these limitations, future research could address these limitations by employing larger and more diverse samples across multiple regions or countries to examine contextual variations in digital adoption. Longitudinal research designs would be particularly valuable for capturing the evolution of behavioral intentions, technology use, and innovation outcomes over time. Furthermore, future studies could explore the integration of advanced digital technologies, such as artificial intelligence (AI) and the Internet of Things (IoT), in upcycling production processes, as well as investigate the role of collaborative networks, policy frameworks, and knowledge-sharing mechanisms in accelerating sustainable digital transformation within micro and small creative enterprises.

## Disclosure statement

No potential conflict of interest was reported by the author(s).

## Author Contributions

Conceptualization: Faza Wahmuda, Ellya Zulaikha, Hadziq Fabroyir

Methodology: Faza Wahmuda, Ellya Zulaikha

Data Curation: Faza Wahmuda

Formal Analysis: Faza Wahmuda

Visualization: Faza Wahmuda, Ellya Zulaikha, Hadziq Fabroyir

Writing – Original Draft Preparation: Faza Wahmuda

Writing – Review & Editing: Ellya Zulaikha, Hadziq Fabroyir

Supervision: Ellya Zulaikha, Hadziq Fabroyir

## Data Availability

Zenodo. Extended Data: Interview Guide for Driving Sustainable Innovation through Digital Transformation. https://doi.org/10.5281/zenodo.17120762 ( Wahmuda et al., 2025). This project contains the following extended data:
• Interview Guide – a structured set of open-ended questions formulated to explore respondents’ experiences, perceptions, and behaviors related to digital transformation and sustainable innovation. The guide ensures consistency during qualitative data collection while allowing respondents the flexibility to articulate their views in their own words. • Construct Measurement Items – a comprehensive set of validated indicators used to assess key constructs in the study, including internal and external factors, perceived usefulness, perceived ease of use, behavioral attitudes, social influences, user experience, digital technology implementation, and innovation roles. These measurement items support the quantitative component of the study by ensuring reliability and alignment with established theoretical frameworks. All data and extended materials are available under the terms of the Creative Commons Attribution 4.0 International (CC BY 4.0) license.
