# Supplementary material for: Driving Sustainable Innovation through Digital Transformation: A Behavioural Model of the Upcycling Craft Industry in Indonesia
Source: F1000Res. 2026 May 5;14:1473. Originally published 2025 Dec 30. [Version 2] doi: 10.12688/f1000research.174640.2 (PMC13237546; doi:10.12688/f1000research.174640.2)
Supplement: Peer review history

# Reviewer report: Reviewer response for version 2

**DOI:** 10.5256/f1000research.196465.r490106

**Published:** 2026-06-04

**Authors:** Benny Hutahayan

**Affiliations:** 1 University of Brawijaya, Malang, East Java, Indonesia

I appreciate that this study uses a mixed-methods approach, but the quantitative section has weaknesses that need to be addressed.  

 I found a research question for a qualitative approach, but the researcher did not mention any hypotheses. The researcher needs to explain how the hypotheses for the quantitative study were formulated and explicitly state them in the article.

 This study uses a Likert scale and cross-sectional data for the quantitative portion; however, it does not test for common method bias, so the researcher should include this analysis. Use Harman’s single-factor test.  

 The results in the results section are rather weak. Validity and reliability have not been adequately tested. First, the researchers cited Hair et al. (2019), but I could not find any factor loadings to assess validity.

 The researchers merely state that the HTMT met the threshold; this is not sufficient. The researchers must present the HTMT results in a table. They should also include the results from Fornell & Larcker to strengthen their argument.

 The researchers have not yet included a VIF test.

 I also did not find the effect size (f2). The researchers need to include it. Bootstrap is also not mentioned in the results.

 Display the R² and Adjusted R² values in the table.

 The researcher should include SRMR, NFI, and PLS Predict (the researcher should include these because SEM analysis was used).

Is the work clearly and accurately presented and does it cite the current literature?

Partly

If applicable, is the statistical analysis and its interpretation appropriate?

Partly

Are all the source data underlying the results available to ensure full reproducibility?

Partly

Is the study design appropriate and is the work technically sound?

Partly

Are the conclusions drawn adequately supported by the results?

Partly

Are sufficient details of methods and analysis provided to allow replication by others?

Partly

Reviewer Expertise:

Business Administration, Human Resources Management, Law, and Governance

I confirm that I have read this submission and believe that I have an appropriate level of expertise to confirm that it is of an acceptable scientific standard, however I have significant reservations, as outlined above.

# Reviewer report: Reviewer response for version 2

**DOI:** 10.5256/f1000research.196465.r481854

**Published:** 2026-05-26

**Authors:** Naimatullah Shah

**Affiliations:** 1 Government of Khyber Pakhtunkhwa Department of Higher Education Archives and Libraries, Peshawar, Pakistan

I congratulate the authors for their outstanding response and efforts to address the comments made in the previous review.

 Changes to Version 2 are significant and very effective. The authors have raised the level of clarity, nuance, and academic rigor in this paper by carefully tempering their claims, explicitly stating the small-sample quantitative phase as an exploratory path, and enhancing the theoretical boundaries of the model of Behavioral-Based Digital Transformation (BBDT).

 The manuscript now presents a beautifully balanced and realistic view of the early stage of digital integration in upcycling micro-enterprises in Indonesia with limited resources.

 The story moves smoothly, the repetitions have been carefully edited, and the restrictions are dealt with in a commendable academic way. 

 I am completely satisfied with this revised version and highly recommend it for indexing.

Is the work clearly and accurately presented and does it cite the current literature?

Yes

If applicable, is the statistical analysis and its interpretation appropriate?

Partly

Are all the source data underlying the results available to ensure full reproducibility?

Partly

Is the study design appropriate and is the work technically sound?

Yes

Are the conclusions drawn adequately supported by the results?

Yes

Are sufficient details of methods and analysis provided to allow replication by others?

Partly

Reviewer Expertise:

I hold a PhD in digital data sets management and have published extensively in library and information management, employing models such as TAM and TPB, as well as bibliometric studies in reputable journals.

I confirm that I have read this submission and believe that I have an appropriate level of expertise to confirm that it is of an acceptable scientific standard.

# Reviewer report: Reviewer response for version 1

**DOI:** 10.5256/f1000research.192561.r447110

**Published:** 2026-01-24

**Authors:** Dwinita Larasati

**Affiliations:** 1 Institut Teknologi Bandung, Bandung, West Java, Indonesia

The scope of "craft industry in Indonesia" is quite extensive and closely related to the main (local) materials. However, this article does not provide any information regarding the materials that are used by the craft industries that are featured as respondents in this research. Meanwhile, an "upcycling" ecosystem and its stakeholders' behavior would depend on the complexity of the materials.

 It would be better if this information is available, in order to be able to identify the "upcycling" possibilities and constraints, which would become factors of consideration within the context of "digital transformation".

Is the work clearly and accurately presented and does it cite the current literature?

Yes

If applicable, is the statistical analysis and its interpretation appropriate?

I cannot comment. A qualified statistician is required.

Are all the source data underlying the results available to ensure full reproducibility?

Partly

Is the study design appropriate and is the work technically sound?

Yes

Are the conclusions drawn adequately supported by the results?

Yes

Are sufficient details of methods and analysis provided to allow replication by others?

Yes

Reviewer Expertise:

design for sustainability, material exploration, design thinking, design business, community development through creative industries, creative economy, creative city

I confirm that I have read this submission and believe that I have an appropriate level of expertise to confirm that it is of an acceptable scientific standard, however I have significant reservations, as outlined above.

# Response

**Published:** 2026-02-11

**Authors:** Ellya Zulaikha

**Affiliations:** Industrial Design Department, Institut Teknologi Sepuluh Nopember, Surabaya, East Java, Indonesia

**Review :**

 The scope of "craft industry in Indonesia" is quite extensive and closely related to the main (local) materials. However, this article does not provide any information regarding the materials that are used by the craft industries that are featured as respondents in this research. Meanwhile, an "upcycling" ecosystem and its stakeholders' behavior would depend on the complexity of the materials.

 It would be better if this information is available, in order to be able to identify the "upcycling" possibilities and constraints, which would become factors of consideration within the context of "digital transformation".    

**Response :**

 Thank you for your input. We have made the following improvements to the chapter and paragraph:

 Section 3. Method 

 Sub section 3.1 – Qualitative Phase

 Paragraph 2 “The three cases were selected not only to represent different stages of digital adoption, but also to capture variation in upcycled material characteristics, encompassing both organic and inorganic waste streams, namely seashell waste (MS), corn cobs (KIK), and glass waste for beaded accessories (BF). This material heterogeneity is analytically relevant because different materials impose distinct production constraints, degrees of processing complexity, and levels of compatibility with digital tools such as CNC machining, laser cutting, or digital design software. Consequently, material characteristics influence the feasible scope and pace of digital integration across the upcycling workflow.”

 Sub section 4.1 A – Case Overview

 Paragraph 1  MS…… “The hardness and structural consistency of seashell waste enabled effective integration with CNC machining and laser engraving, allowing higher precision and repeatability.”…….

 Paragraph 2 KIK….”As an organic and heterogeneous material, corn cobs present higher variability in texture, density, and durability, which requires iterative prototyping and limits the full automation of digital fabrication processes.”…..

 Paragraph 3 BF…..”Glass waste used for beaded accessories is characterized by fragility and small-scale manual assembly requirements, which constrain the applicability of digital fabrication technologies beyond design visualization and digital marketing activities.”…..

# Reviewer report: Reviewer response for version 1

**DOI:** 10.5256/f1000research.192561.r449263

**Published:** 2026-01-24

**Authors:** Naimatullah Shah

**Affiliations:** 1 Government of Khyber Pakhtunkhwa Department of Higher Education Archives and Libraries, Peshawar, Pakistan

The manuscript addresses a timely and relevant topic at the intersection of digital transformation, behavioral theory, and circular economy practices in micro-scale creative enterprises. The qualitative insights are rich and contextually grounded, and the study contributes empirical evidence from an under-researched sector and geographical context.

 However, despite these strengths, the manuscript presents several methodological, theoretical, and analytical limitations that significantly constrain the robustness, generalizability, and theoretical contribution of the findings. The issues mentioned below should be carefully addressed to strengthen the manuscript.

 1. The quantitative phase relies on a sample of 30 respondents, which is very limited for testing a multi-construct structural equation model with mediation effects.  The authors should acknowledge this limitation more critically, and clearly position the quantitative results as exploratory rather than confirmatory.

 2. The study adopts a cross-sectional design in both qualitative and quantitative phases. Digital transformation and behavioral intention are inherently dynamic and evolutionary processes, yet the research design does not capture changes over time, learning trajectories, or post-adoption outcomes. Claims related to “transformation” should be moderated, and future research directions should more strongly emphasize longitudinal designs.

 3. The construct *“Attitude and Intention toward Digital Adoption”* combines two theoretically distinct elements from TAM and TPB. This aggregation: a). Reduces conceptual clarity, b). Inflates mediation effects, c). Obscures the specific role of attitudes versus intentions. The authors should justify this aggregation.

 4. The study is narrowly focused on Indonesian upcycling craft micro-enterprises, yet some implications are presented in broader SME or creative industry terms. The authors should more clearly delimit the contextual scope of their conclusions.

 5. The manuscript is lengthy, with some repetition across the literature review and discussion sections.

 6. Several sections reiterate TAM–TPB arguments without adding new analytical insight.

Is the work clearly and accurately presented and does it cite the current literature?

Yes

If applicable, is the statistical analysis and its interpretation appropriate?

Partly

Are all the source data underlying the results available to ensure full reproducibility?

Partly

Is the study design appropriate and is the work technically sound?

Yes

Are the conclusions drawn adequately supported by the results?

Yes

Are sufficient details of methods and analysis provided to allow replication by others?

Partly

Reviewer Expertise:

I hold a PhD in digital data sets management and have published extensively in library and information management, employing models such as TAM and TPB, as well as bibliometric studies in reputable journals.

I confirm that I have read this submission and believe that I have an appropriate level of expertise to confirm that it is of an acceptable scientific standard, however I have significant reservations, as outlined above.

# Response

**Published:** 2026-02-11

**Authors:** Ellya Zulaikha

**Affiliations:** Industrial Design Department, Institut Teknologi Sepuluh Nopember, Surabaya, East Java, Indonesia

**Reviewer's Comment (1) :**

 The quantitative phase relies on a sample of 30 respondents, which is very limited for testing a multi-construct structural equation model with mediation effects.  The authors should acknowledge this limitation more critically, and clearly position the quantitative results as exploratory rather than confirmatory.    

**Response :**

 Thank you for this constructive comment. We acknowledge that the quantitative phase, based on a sample of 30 respondents, does not permit confirmatory testing of a multi-construct structural equation model with mediation effects. Accordingly, we have clarified the exploratory and predictive nature of the quantitative analysis and more explicitly positioned the results as preliminary rather than confirmatory. The manuscript has been revised as follows:

 Sub section 3.2 – Quantitative Phase

 Paragraph 1 “The second phase of the study aims to address RQ3 by examining the relationships among the variables and providing exploratory empirical support for the proposed BBDT model.” 

 Paragraph 4 “Given the limited sample size (n = 30), the application of PLS-SEM in this study should be interpreted as exploratory and predictive hypothesis testing rather than confirmatory analysis. As noted by Hair et al. (2014; 2019), PLS-SEM is particularly suitable for exploratory research and studies with relatively small sample sizes. Accordingly, the statistical findings provide preliminary evidence of the proposed relationships, indicating the need for replication with larger samples to strengthen and complement the qualitative findings through data triangulation.”

 Sub section 4.2 – Quantitative Results

 Paragraph 6 “Given the exploratory nature of the analysis and the limited sample size, the statistical relationships identified in this study should be interpreted as indicative patterns rather than definitive causal effects.”

 Section 6 – Conclusion, Limitation, and Future Research

 Paragraph 3 “Despite these contributions, this study has limitations. The sample size in the quantitative phase was relatively small and limited to select regions in Indonesia, which may constrain the generalizability of the findings. Accordingly, the quantitative findings are limited to providing exploratory insights rather than confirmatory evidence and primarily function to complement the triangulation of the qualitative findings. Consequently, the quantitative results cannot yet be fully generalized, and the application of a purely quantitative approach may yield different findings.”

**Reviewer's Comment (2) :**

 The study adopts a cross-sectional design in both qualitative and quantitative phases. Digital transformation and behavioral intention are inherently dynamic and evolutionary processes, yet the research design does not capture changes over time, learning trajectories, or post-adoption outcomes. Claims related to “transformation” should be moderated, and future research directions should more strongly emphasize longitudinal designs.

**Response :**

 Thank you for highlighting this important methodological issue. We acknowledge that the study adopts a cross-sectional design in both the qualitative and quantitative phases and therefore cannot capture temporal dynamics, learning trajectories, or post-adoption outcomes. We have moderated the claims related to “transformation” and strengthened the emphasis on early-stage processes and the need for longitudinal research. The manuscript has been revised as follows:

 Sub section 3.2 – Quantitative Phase

 Paragraph 6 “Given the cross-sectional nature of the study, digital transformation is conceptualized as an early-stage behavioral and technological pathway rather than a fully observed longitudinal organizational change. This conceptualization is consistent with prior digital transformation frameworks that distinguish initial readiness or digitization stages from deeper process and business model transformation (Verhoef et al., 2021; Tian & Ou, 2024; Jia et al., 2024).”

 Sub section 4.2 – Quantitative Results

 Paragraph 6 “Accordingly, the observed relationships should be interpreted as capturing early-stage digital adoption outcomes and transformation-relevant behaviors, rather than mature or fully institutionalized digital transformation. This interpretation aligns with multi-stage digital transformation models, which characterize initial phases as focused on readiness formation, limited implementation depth, and exploratory integration of digital tools (Berghaus & Back, 2017; Verhoef et al., 2021; Tian & Ou, 2024).”

 Sub section 5.1 – Discussion

 Paragraph 8 “Importantly, the findings should be situated within the broader literature on early-stage digital transformation. Prior studies consistently distinguish initial phases of digital transformation—often labeled as digitization, initial readiness, or fuzzy front-end stages—from deeper organizational and business model transformation (Berghaus & Back, 2017; Leipzig et al., 2017; Verhoef et al., 2021). In these early phases, organizations typically focus on exploratory adoption, foundational capability building, and the formation of digital orientation rather than on fully integrated transformation outcomes.

 The present study aligns with this perspective by capturing how behavioral readiness, attitudes, and intentions shape the initiation and early implementation of digital technologies in micro-scale upcycling enterprises. Similar to findings in SMEs and other resource-constrained organizations, external pressures and perceived usability tend to dominate early-stage adoption, while organizational structures and advanced digital capabilities become more influential in later stages (Jia et al., 2024; Zhang et al., 2025). Thus, the BBDT model should be interpreted as explaining early-stage behavioral pathways that enable digital transformation to begin, rather than as a model of mature or completed transformation.”

 Section 6 - Conclusion, Limitation, and Future Research

 Paragraph 3 “In addition, the cross-sectional design limits the ability to observe progression across digital transformation stages. Consistent with multi-stage digital transformation frameworks, future research should adopt longitudinal designs to examine how early-stage behavioral readiness and initial digital adoption evolve into deeper process integration and organizational transformation over time (Verhoef et al., 2021; Tian & Ou, 2024).”

**Reviewer's Comment (3) :**

 The construct “Attitude and Intention toward Digital Adoption” combines two theoretically distinct elements from TAM and TPB. This aggregation: 

 a)    Reduces conceptual clarity, 

 b)    Inflates mediation effects, 

 c)    Obscures the specific role of attitudes versus intentions. 

 The authors should justify this aggregation.    

**Response :**

 Thank you for this insightful comment. We acknowledge that attitude and intention are conceptually distinct constructs in TAM and TPB. However, we have clarified and strengthened the justification for their aggregation in the specific context of owner-managed micro-enterprises and have also acknowledged the associated limitations. The manuscript has been revised as follows:

 Sub section 3.2 – Quantitative Phase

 Paragraph 2 “In the context of owner–operator micro-enterprises, the owner typically acts simultaneously as founder, manager, and primary decision-maker. As a result, attitudinal evaluation and intention formation regarding digital adoption are often enacted by the same individual and within a single decision episode. Prior studies on micro-enterprises and sole proprietorships indicate that owners’ attitudes, aspirations, and personal predispositions directly translate into adoption decisions, making empirical separation between attitude and intention less distinct in practice (Jones et al., 2014; Vatanasakdakul et al., 2020; Nair et al., 2019). Accordingly, this study operationalizes attitude and intention as a composite construct to capture the owner–operator’s overall digital adoption orientation.”

 Sub section 4.2 – Quantitative Results

 Paragraph 6 “In particular, the strong mediating effects observed for the combined Attitude and Intention construct reflect overall behavioral readiness toward digital adoption rather than distinct attitudinal or intentional mechanisms. 

 Sub section 5.1 – Discussion 

 Paragraph 6 “While TAM and TPB conceptually distinguish between attitude and intention, empirical studies in micro-enterprises and MSMEs consistently show that attitude strongly and directly shapes intention, particularly when decision-making authority is highly centralized in the owner–operator. In such contexts, attitude and intention tend to operate as an integrated behavioral orientation rather than as sequentially separable constructs (Vatanasakdakul et al., 2020; Jones et al., 2014; Rahmawaty et al., 2025; Adu et al., 2022). The present findings align with this stream of research, suggesting that behavioral readiness in micro-scale upcycling enterprises reflects a unified cognitive–motivational stance toward digital adoption.”

 Section 6. - Conclusion, Limitation, and Future Research

 Paragraph 3 “Another limitation concerns the aggregation of attitude and intention into a single construct. While this operationalization is contextually justified for owner-managed micro-enterprises, it limits analytical granularity and may obscure potential sequential effects between attitude formation and intention enactment. Future studies should disentangle these constructs to examine differential behavioral pathways using larger samples and longitudinal designs.” 

**Reviewer's Comment (4) :**

 The study is narrowly focused on Indonesian upcycling craft micro-enterprises, yet some implications are presented in broader SME or creative industry terms. The authors should more clearly delimit the contextual scope of their conclusions.    

**Response :**

 Thank you for this important clarification. We acknowledge that the empirical focus of the study is limited to Indonesian upcycling craft micro-enterprises and that broader generalizations should be avoided. We have therefore explicitly delimited the contextual scope of the conclusions in the manuscript, as reflected in the following revision:

 Section 6. - Conclusion, Limitation, and Future Research

 Paragraph 2 “These implications should be interpreted within the context of Indonesian upcycling craft micro-enterprises, where resource constraints, informal work structures, and owner-centered decision-making may differ from larger SMEs or other creative sectors.”

**Reviewer's Comment (5) :**

 The manuscript is lengthy, with some repetition across the literature review and discussion sections.

**Response :**

 Thank you for this helpful observation. We acknowledge that the manuscript contained some repetition, particularly between the literature review and the discussion sections. We have revised the literature review to reduce redundancy, streamline overlapping arguments, and improve concision, especially in:

 Section 2.    Literature Review

 Sub Section 2.1.    Behavioral Foundations of Digital Transformation in Micro Creative Enterprises

 Paragraph 1 “Behavioral foundations ……Classical technology adoption theories………evaluations and social pressures shape adoption behavior.”

 Paragraph 2 “When situated within the context ……”

 Paragraph 3 “Despite growing scholarly …….”

 Paragraph 4 “In parallel, Prieto-Sandoval et al. (2021) …

 Paragraph 5 “Synthesizing these insights…..

**Reviewer's Comment (6) :**

 Several sections reiterate TAM–TPB arguments without adding new analytical insight.    

**Response :**

 Thank you for pointing this out. We agree that several sections previously reiterated TAM–TPB arguments without sufficient additional analytical contribution. We have revised the discussion to emphasize context-specific insights and empirical contributions beyond the standard TAM–TPB framework. In particular:

 Sub section 5.1 Discussion

 Paragraph 3 “Unlike prior TAM–TPB studies predominantly conducted in larger SMEs or formal organizational settings, the findings of this study suggest that in micro-scale upcycling enterprises behavioral intention is closely tied to immediate production experiences and material outcomes rather than abstract performance expectations. Perceived usefulness and ease of use are not evaluated separately, but emerge through hands-on experimentation, iterative learning, and direct engagement with digital tools in everyday production practices.”
